# POT1 tumour predisposition: a broader spectrum of associated malignancies and proposal for additional screening program

**DOI:** 10.1038/s41431-024-01611-0

**Published:** 2024-06-05

**Authors:** Marta Baptista Freitas, Laurence Desmyter, Cindy Badoer, Guillaume Smits, Isabelle Vandernoot, Daphné t´Kint de Roodenbeke

**Affiliations:** 1 Centro Hospitalar Universitário de São João, Oporto, Portugal; 2 Center for Human Genetics, Hôpital Erasme, Hôpital Universitaire de Bruxelles, Université Libre de Bruxelles, Brussels, Belgium; 3 Department of Genetics, Hôpital Universitaire Des Enfants Reine Fabiola, Hôpital Universitaire de Bruxelles, Université Libre de Bruxelles, Brussels, Belgium; 4 Jules Bordet Institute, Université Libre de Bruxelles, Brussels, Belgium

**Keywords:** Cancer genetics, Genetics research

## Abstract

Protection of Telomeres Protein 1 (POT1) protein is an essential subunit of the shelterin telomere binding complex, regulating telomere length. Some *POT1* gene pathogenic variants (PV) lead to telomere elongation, genomic instability and higher risk of cancer. *POT1* tumour predisposition syndrome (POT1-TPD) has autosomal dominant inheritance and unknown penetrance. It is associated with increased risk of cutaneous melanoma, chronic lymphocytic leukaemia, angiosarcoma and gliomas. In this work, we aim to describe a broader cancer phenotype related to POT1-TPD, in three families (two with a four generation pedigree, one with a five generation pedigree). The three index cases were referred to our oncogenetic centre for genetic counselling due to their personal history of cancer. Two underwent clinical exome sequencing of 4,867 genes associated with Mendelian genetic diseases, and another underwent gene panel sequencing including *POT1*, which identified three different *POT1* PV: NC_000007.14(NM_015450.2):c.349C>T; NC_000007.14(NM_015450.2):c.233T>C and NC_000007.14(NM_015450.2):c.818G>A; already described in the literature. Referenced relatives, did a target genetic test (according to the *POT1* PV identified in the family). In total, 37 individuals were tested (51.4% females), median age of 46 (22–81) years, with *POT1* PV detected in 22. POT1-TPD was observed, but also a higher incidence of other cancers (other sarcomas, papillary thyroid cancer, early onset prostate cancer and leukaemia). These findings contribute to an increase in our knowledge about *POT1* PV, and it can play a role in the definition of future *POT1* PV screening criteria, *POT1* carrier surveillance protocols (possibly considering screening for all types of sarcomas) and in genetic counselling.

## Introduction

Protection of Telomeres Protein 1 [1] gene is located on chromosome 7 (7q31.33) and *POT1* protein is an essential subunit of the shelterin telomere binding complex [1, 2]. It binds to the telomeric overhangs, preventing the activation of DNA damage response at telomeres and regulating telomere length [2, 3]. *POT1* pathogenic variants (PV) can present with different phenotypes [3]. Some variants cause telomere truncations leading to dysfunctional telomeres and causing a telomeric syndrome known as Coats Plus Syndrome, others cause telomere shortening that leads to as idiopathic pulmonary fibrosis [3]. On the other hand, there are *POT1* variants that cause an opposite telomere phenotype, allowing telomere elongation which leads to prolongation of cell lineage’s life span, facilitating the acquisition of several somatic mutations [4, 5]. This loss of the tumour-suppressor mechanism of telomere shortening leads to clonal population expansion and genomic instability, predisposing to a higher risk of cancer [3, 6].

*POT1* tumour predisposition syndrome (POT1-TPD) is inherited in an autosomal dominant manner and associated with an increased lifetime risk of cutaneous melanoma (CM), chronic lymphocytic leukaemia (CLL), angiosarcoma (mostly cardiac angiosarcomas) and gliomas [1–3, 7–13]. Other types of cancer, such as colorectal cancer (CRC), thyroid cancer, other soft tissue sarcomas (STS), osteosarcomas, breast and lung cancers have also been associated with POT1-TPD [8, 11, 14–20]. Moreover, *POT1* variant carriers seem to have a higher risk of B-cell and T-cell lymphoproliferative and myeloproliferative disease [6]. However, these other types of malignancies are not yet considered in the surveillance recommendations for POT1-TPD carriers [1].

The penetrance of POT1-TPD is currently unknown, since only several hundred probands have been tested [1, 13]. Therefore, the full phenotypic spectrum and penetrance of this syndrome is yet to be determined [1, 3].

POT1-TPD diagnosis is made using a molecular genetic test, with the detection of a heterozygous germline PV in the *POT1* gene [1]. It should be suspected in people with multiple CM, one of POT1-TPD core cancers (CM, CLL, angiosarcoma or glioma) and a first- or second-degree relative with a confirmed POT1-PTD cancer, or a somatic *POT1* PV identified on tumour tissue sequencing [1].

There is no targeted treatment available for *POT1* PV. POT1-TPD is treated according to the standard of care for each type of tumour [1, 2]. However, longer telomeres and upregulation of *POT1* were associated with resistance to radiotherapy, in cell line studies [21–23]. Therefore, the development of *POT1* and telomerase inhibitors may be a potential approach to enhance radiosensitivity in these tumours [23].

Most of the tumours related to *POT1* PV are diagnosed in adulthood [1, 3]. The age of onset for first primary tumour described is 15 years [12], therefore all the surveillance procedures are recommended to start at age 18 or two to five years earlier than the earliest diagnosis in the family [1]. There are no published guidelines for surveillance of *POT1* PV carriers, the following recommendations are based on an expert opinion publication about POT1-TPD that also addresses this topic [1]. For *POT1* mutation carriers, a comprehensive physical examination with careful annual examination of lymph nodes, full skin examination by a dermatologist (every three to six months in individuals with multiple atypical naevi, personal history of CM, and/or family history of CM) and an annual complete blood count is recommended [1]. In families with Li–Fraumeni syndrome or Li–Fraumeni-like criteria, an annual whole-body MRI is recommended [1]. It can also be considered in other carriers depending on personal and family history of non-cutaneous and non-brain malignancies (every one to two years) [1]. In families with glioma cases, a brain MRI every one to two years is recommended [1].

With this work, we aim to describe all types of tumours diagnosed in three families carrying a *POT1* PV, both malignant diseases previously described as associated with POT1-TPD and also other cancer types not described or less often reported as part of this syndrome, showing a broader phenotype of *POT1* associated tumours.

## Materials and methods

Probands were referred to our oncogenetic centre, *Jules Bordet Institute*, in Brussels, Belgium. Cancer diagnoses were confirmed by pathological specimen review (in index cases of families A and B), medical records (in index case of family C – previously diagnosed and treat in another medical centre; and in all the tested relatives from families A, B and C), detailed direct anamnesis and also based on self or family report. All patients signed an informed consent for genetic testing.

We performed for index cases of families A and B a clinical exome sequencing of 4867 genes associated with mendelian genetic diseases, filtered to analyse a panel of cancer susceptibility genes. Index case of family C did a gene panel sequencing, which included *POT1*.

Relatives referenced to our centre, did a target gene analysis, which was performed with PCR amplification followed by direct Sanger sequencing of exon 8/7/10 (according to the mutation found in the family) of *POT1* gene. Our reference sequence is the coding sequence NM_015450 (A of ATG = 1). In all cases, a control of the result was made on an independent sample.

All tested individuals were evaluated through a genetic counselling consultation and received pre-genetic testing psychological support.

## Results

In total, 37 individuals were tested: 19 females and 18 males, median age of 46 (22–81) years, between 2019 and 2023.

### Family A

**Fig. 1:**
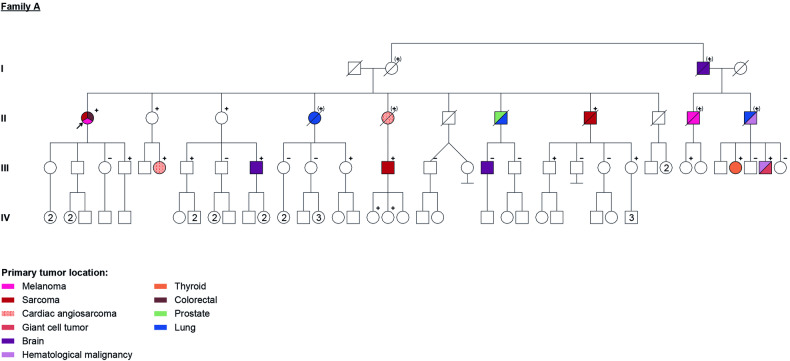
Family A - four generation pedigree with several family members affected with different primary cancers. Family members with a cancer diagnosis are shown as filled symbol. All tested family members are marked in the pedigree, others were not tested. + : positive for NC_000007.14(NM_015450.2):c.349C>T variant; − : negative for NC_000007.14(NM_015450.2):c.349C>T variant; (+) : obligate carrier of NC_000007.14(NM_015450.2):c.349C>T variant.

The index case (Fig. 1, II.1) is a female patient, with Ashkenazi Jewish ancestry, a history of right arm sarcoma at age 65, cutaneous melanoma (CM) at age 66 and colon cancer at age 67. Among first degree family members there was history of lung cancer at age 63 (Fig. 1, II.4), early cardiac angiosarcoma at age 28 (Fig. 1, II.5), prostate cancer at unknown age, but before 70 years old (Fig. 1, II.7), lung cancer at age 77 (Fig. 1, II.7) and tibial osteosarcoma at age 49 (Fig. 1, II.8). After genetic counselling, the patient benefited from a genetic test that detected a PV in *POT1* gene: NC_000007.14(NM_015450.2):c.349C>T (Table 1).

**Table 1:** Germline variants shared by the affected individuals that were analysed according to their family.

Family	Gene	Transcript	Chr	Position	Exon	Codon	Protein	Molecular consequence	Variant Classification
A	*POT1*	NM_015450.2	7	124503601	8	c. 349C>T	p. (Arg117Cys)	Missense	Likely Pathogenic
B	*POT1*	NM_015450.2	7	124510987	7	c. 233T>C	p. (Ile78Thr)	Missense	Likely Pathogenic
C	*POT1*	NM_015450.2	7	124493077	10	c. 818G>A	p. (Arg273Gln)	Missense	Likely Pathogenic

*Chr* chromosome.

Regarding second- and third-degree relatives, there were: one individual with cardiac angiosarcoma at age 29 (Fig. 1, III.6), two individuals with brain tumours – glioblastoma at age 50 (Fig. 1, III.16) and another with undefined histology at unknown age (Fig. 1, I.3); one individual with testicular leiomyosarcoma at age 45 (Fig. 1, III.13).

Regarding more distant relatives, there was history of a CM at age 42 (Fig. 1, II.10), a lung cancer at age 78 (Fig. 1, II.11), a leukaemia at age 78 (Fig. 1, II.11), a papillary thyroid cancer (PTC) at age 37 (Fig. 1, III.28), a giant cell tumour of bone at age 40 (Fig. 1, III.30) and a CLL at age 45 (Fig. 1, III. 30). The relative III.25 has history of multiple breast fibroadenoma.

In total, genetic tests were performed on twenty-seven family relatives (twenty-one of them as pre-symptomatic tests). The NC_000007.14(NM_015450.2):c.349C>T variant was detected in sixteen of them (eleven without history of cancer at the time of the test). Moreover, six of the non-tested individuals (Fig. 1, I.2, I.3, II.4, II.5, II.10 and II.11) are obligate carriers of the mutation, since at least one of their children tested positive.

The individual III.9 was diagnosed with low grade glioma (at age 50) after brain MRI, prescribed as part of the screening program for *POT1* carriers.

The individual III.16, besides having a glioblastoma had a negative test for the *POT1* familiar variant.

### Family B

**Fig. 2:**
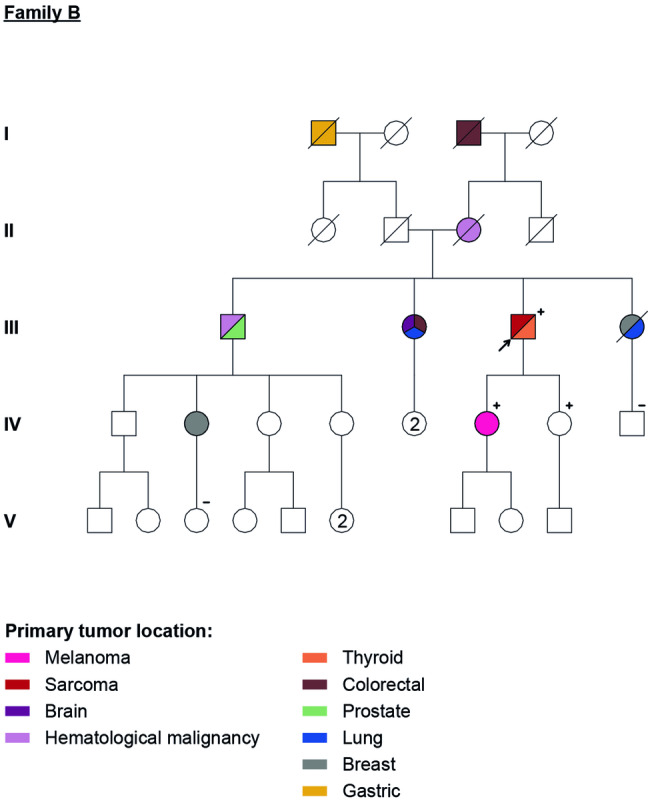
Family B - five generation pedigree with multiple family members affected with different primary cancers. Family members with a cancer diagnosis are shown as filled symbol. All tested family members are marked in the pedigree, others were not tested. + : positive for NC_000007.14(NM_015450.2):c.233T>C variant; − : negative for NC_000007.14(NM_015450.2):c.233T>C variant; (+) : obligate carrier of NC_000007.14(NM_015450.2):c.233T>C variant.

The index case (Fig. 2, III.3) is a male patient, also with Ashkenazi Jewish ancestry, with history of left leg and arm liposarcomas at age 73, a PTC at age 74. Regarding first degree family members, there was history of two leukaemia at age 70 (Fig. 2, II.3) and 83 (Fig. 2, III.1), one CM at age 30 (Fig. 1, IV.7), one prostate cancer at age of 60 (Fig. 2, III.1); one relative with colon carcinoma at age 65, lung cancer at age 81 and brain tumour at an unknown age of diagnosis (Fig. 2, III.2); and another relative with breast cancer at age of 44 and lung cancer at the age of 68 (Fig. 2, III.4). The genetic testing of the index patient detected a PV in *POT1* gene: NC_000007.14(NM_015450.2):c.233T>C (Table 1).

Among second- and third-degree relatives, there were: one gastric cancer at 65 years (Fig. 2, I.1), one colon cancer at age 60 (Fig. 2, I.3) and one breast cancer at age 43 (Fig. 2, IV.2).

In total, four other relatives were tested (pre-symptomatic testing for three of them). The NC_000007.14(NM_015450.2):c.233T>C variant was detected in the two daughters of the proband, one of them having history of CM (Fig. 2 IV.7).

### Family C

**Fig. 3:**
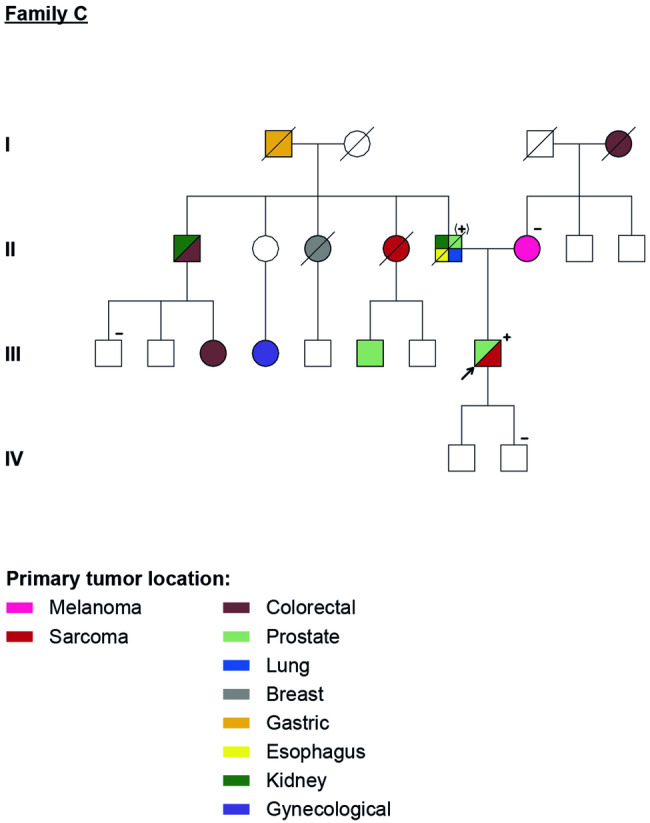
Family C - four generation pedigree with several family members affected with different primary cancers. Family members with a cancer diagnosis are shown as filled symbol. All tested family members are marked in the pedigree, others were not tested. + : positive for NC_000007.14(NM_015450.2):c.818G>A variant; − : negative for NC_000007.14(NM_015450.2):c.818G>A variant; (+) : obligate carrier of NC_000007.14(NM_015450.2):c.818G>A variant.

The index case (Fig. 3, III.8) is a male patient, with a history of prostate cancer at age 47 and an undifferentiated spindle cell sarcoma of the left arm at age 57. The patient had previously undergone genetic tests in 2009 and 2011 in other institutions with a panel of several genes (including *BRCA1, BRCA2, PTEN, CHEK2, TP53* as well as Lynch syndrome genes) without any anomaly detected. Considering his personal (a second cancer diagnosis – arm sarcoma) and family history, an additional genetic analysis was proposed in 2022 in order to search for mutations in other genes, in particular on *POT1* whose spectrum could correspond to the history of cancers reported and was included in our oncogenetic gene panel. This third genetic test detected a *POT1* pathogenic variant: NC_000007.14(NM_015450.2):c.818G>A (Table 1).

Regarding the maternal side of the family, only two cases with oncological history (first- and second-degree relatives) and in older ages were present: one CM at age 72 (Fig. 3, II.6) and one colon cancer at age 70 (Fig. 3, I.4). The mother was tested and *POT1* mutation was not detected. Several members had a history of cancer on the paternal side: a gastric cancer at unknown age of diagnosis (Fig. 3, I.1), two kidney cancers (Fig. 3, II.1 – at age 54, and II.5 – at age 60), two colon cancers (Fig. 3, II.1 – after 65 years old, and Fig. 3, III.3 – unknown age), a breast cancer after 50 years old (Fig. 3, II.3), a left leg STS at age of 40 (Fig. 3, II.4), two early onset prostate cancers (Fig. 3, II.5 – under 60 years old, and III.6 at age 40), an oesophageal cancer at unknown age of diagnosis (Fig. 3, II.5), a lung cancer at age 70 (Fig. 3, II.5) and a gynaecological cancer at unknown age (Fig. 3, III.4). Despite this frequent cancer incidence on the paternal side of the family, only one of these relatives was pre-symptomatically tested (Fig. 3, III.1), and had a negative result.

Another relative, also pre-symptomatic, was tested (Fig. 3, IV.2), with a negative result.

Table 2 summarizes the types of tumours in these tree families, according to *POT1* status and Table 3 specifies the age of onset of each tumour per individual.

**Table 2:** Tumour types of different members of the studied families according to *POT1* status.

In grey background, there are the tumours considered strongly related to POT1 mutations.

*AS* angiosarcoma, *BT* brain tumour, *CM* cutaneous melanoma, *CRC* colorectal cancer, *GCBT* giant cell tumour of bone, *Haem* haematological, *NT* non-tested, *POT1* *pv* POT1 pathogenic variant, *PTC* papillary thyroid carcinoma.

**Table 3:** Tumour types of different members of the studied families according to *POT1* status and age of diagnosis.

	Proband	Tumour (age of diagnosis)
With *POT1* pv	AII.1	Soft tissue sarcoma (65); Cutaneous melanoma (66); CRC (67)
	AII.8	Osteosarcoma (49)
	AIII.6	cardiac angiosarcoma (29)
	AIII.9	Low grade glioma (50)
	AIII.13	Testicular leiomyosarcoma (45)
	AIII.28	PTC (37)
	AIII.30	GCTB; Chronic lymphocytic leukaemia (45)
	BIII.3	2 Liposarcomas (73); PTC (74)
	BIV.7	Cutaneous melanoma (30)
	CIII.8	Prostate cancer (47); USCS (57)
NT individuals - obligated carriers	AI.3	Brain cancer (unk)
	AII.4	Lung cancer (63)
	AII.5	Cardiac angiosarcoma (28)
	AII.10	Cutaneous melanoma (42)
	AII.11	Lung cancer (78) + Leukaemia (78)
	CII.5	Prostate cancer (<60); Kidney cancer (60); Lung cancer (70); EC (unk)
Unknown *POT1* status	AII.7	Prostate cancer (<70); Lung cancer (77)
	BI.1	Gastric Cancer (65)
	BI.3	CCR (60)
	BII.3	Leukaemia (70)
	BIII.1	Prostate cancer (60); Leukaemia (83)
	BIII.2	CRC (65); Brain cancer (unk), Lung cancer (81)
	BIII.4	Breast cancer (44); Lung cancer (68)
	BIV.2	Breast cancer (43)
	CI.1	Gastric cancer (unk)
	CI.4	CRC (70)
	CII.1	Kidney cancer (54); CRC (>65)
	CII.3	Breast cancer (>50)
	CII.4	Soft tissue sarcoma (40)
	CIII.3	CRC (unk)
	CIII.4	Gynaecological cancer (unk)
	CIII.6	Prostate cancer (40)
Without *POT1* pv	AIII.16	Glioblastoma (50)
	CII.6	Cutaneous melanoma (72)

The age of diagnose is written between brackets.

*CRC* colorectal cancer, *EC* esophageal cancer, *GCTB* giant cell tumour of bone, *NT* non-tested, *POT1* pv *POT1* pathogenic variant, *PTC* papillary thyroid carcinoma, *unk* unknown, *USCS* undifferentiated spindle cell sarcoma.

## Discussion

In family A, the NC_000007.14(NM_015450.2):c.349C>T pathogenic variant was identified, which leads to replacement of an arginine with a cysteine at amino acid 117 (p. Arg117Cys) (Table 1). This has already been reported in three Li–Fraumeni like families (with cardiac angiosarcomas and other STS) [24]. These individuals had reduced telomere-bounded *POT1* levels, with longer and more fragile telomeres and one mutation carrier also developed cutaneous melanoma (CM) [24]. In a recent study that analysed more than 1500 cases of sarcoma probands, *POT1* PV was identified in six and associated to familial melanoma pedigree in two of these [19]. Further, another study reported higher prevalence of CM (13.2%) and sarcomas (3.5%) among POT1 PV carries [13]. These findings are in accordance with the observations in family A: index case with STS and CM, two relatives with early onset cardiac angiosarcoma (two with other STS) and another one with CM. Nonetheless, two cases of brain tumours and a case of lymphoproliferative syndrome were identified, which have not been described before in association with this specific *POT1* PV, but are known to be related to POT1-TPD, reinforcing the recognised disease spectrum of *POT1* mutations [1]. On the other hand, one can observe that individuals with the mutation also developed other types of cancers that have been described in association with mutation of *POT1*, but with less evidence, such as colon cancer, lung cancer, leukaemia and PTC [6, 8, 13, 16, 17]. Also, the relative III.30 developed a bone giant cell tumour, which despite not being a malignant tumour is clinically relevant, and was not previously associated with *POT1* PV. Surprisingly, one case of glioblastoma was not related with the familial *POT1* mutation (Fig. 1, III.16), and was thus a phenocopy. This phenomenon is more likely to occur in large families, such as family A.

Regarding family B, we detected the NC_000007.14(NM_015450.2):c.233T>C pathogenic variant, which results in isoleucine replaced by a threonine in *POT1* protein (p. Ile78Thr) (Table 1). This PV has been involved in familial melanoma (three of these families with self-reported Jewish descent as in family B), lymphoid and myeloid neoplasms [6, 25–27]. In family B, there is one case of CM (in a carrier) and two cases of leukaemia (in non-tested relatives), in accordance with the previous descriptions regarding this PV. This family had a broader cancer spectrum: STS, PTC, brain tumour, prostate, colon, lung, gastric and breast cancer, although mostly in non-tested individuals. Therefore, we cannot formally establish a causal relationship between these tumours and the presence of the *POT1* PV. Nonetheless, by analysing the index case it is possible to suppose that this family harbours a broader spectrum of POT1-TPD, since the patient was diagnosed with two STS (other than angiosarcomas) and a PTC, already described as associated with *POT1* mutation, but not related to this specific PV, neither considered in the surveillance recommendations as a POT1-TPD [1, 17, 19].

In family C, NC_000007.14(NM_015450.2):c.818G>A variant in *POT1* gene was detected in the proband (p. Arg273Gln) (Table 1). This variant was already described in association with CM [8] and also related to a higher risk of lymphoid and myeloid clonal haematopoiesis [6]. All predictive in silico tools, that evaluate the effect of missense changes on protein structure and function, suggest that this variant is likely to be disruptive [28]. These arguments, and the good concordance with the phenotype, indicate that the variant is likely to be pathogenic. In family C, there was only one case of CM in a non-carrier (Fig. 3, II.6). Since the mother was negative for the *POT1* mutation, we can hypothesise that this is a case of *de novo POT1* PV or a case of paternal inheritance. This last hypothesis seems the more probable due to the wider spectrum of cancers on that side of the family, although none of those relatives were tested. The tested carrier in this family (Fig. 3, II.5) presented an early onset prostate cancer and a STS, tumours not traditionally associated with POT1-TPD, but already reported in another POT1 related study [13].

In these three families, it was possible to observe the typical POT1-TPD: two cases of CM, two cases of cardiac angiosarcoma, one case of CLL and two cases of brain tumours, in total. Nevertheless, a broader cancer spectrum related to *POT1* mutations is described. It was possible to observe several sarcomas other than angiosarcomas (six in total). As referred before, there is growing evidence suggesting that *POT1* PV could increase the risk of sarcoma and not just angiosarcoma [2, 13, 18, 19, 24]. Regarding CRC and PTC, already mentioned as being associated to POT1-PTD, but not included in the recommendations for screening and surveillance of these patients, here we observed one CRC case and two PTC in carriers, reinforcing this possible association [1, 16, 17]. Another *POT1* PV (p.V29L) was described in a family with PTC [17]. However, it is known that several other hereditary syndromes are associated with differentiated thyroid cancer [29], so further studies are needed regarding *POT1* mutations and the risk of PTC. Concerning POT1-TDP and CRC risk, a study with thousands of patients affected with CRC detected three *POT1* PV in affected individuals, pointing this gene as a candidate for CRC susceptibility genes [16].

Regarding haematological diseases, it is well documented that *POT1* mutations increase CLL risk [8, 12, 30]. Moreover, a recent study showed the relationship between long telomeres due to *POT1* mutations and familial clonal haematopoiesis syndrome, which broadens the spectrum of predisposition to malignant haematological diseases [6]. The p. Ile78Thr variant identified in family B was already described to be associated with CLL and myelodysplastic syndromes in a large study [27]. Indeed, family B has two cases of leukaemia, although in non-tested individuals. A third case of leukaemia was described in family A, in an obligate carrier. Despite the absence of leukaemia reported in association to the p. Arg117Cys variant, considering the recent data on the increased risk of malignant haematological diseases associated to *POT1* mutations [6], we cannot exclude the association between leukaemia and this *POT1* PV. Future studies will be important to investigate the link between specific *POT1* PV and the incidence of haematological diseases.

Lung cancer was observed in six family relatives (including two p. Arg117Cys variant carriers), but there is no description of its association with POT1-TPD. A study with more than 30,000 lung cancer patients showed that *POT1* mutations play a role in lung cancer predisposition [15], however, without further data it is not possible to draw a clear conclusion on the association of *POT1* PV and lung cancer.

Finally, early onset prostate cancer was present in three individuals (one carrier, one obligate carrier and one non-tested). A recent study reported higher prostate cancer among men with *POT1* PV, but with a median age of onset of 67.5 years [13]. Nonetheless, these cases can indicate a possible link that needs further investigation.

Several pre-symptomatic tests were performed on relatives of the three families, with the detection of *POT1* mutations in 16 individuals. All the individuals carrying a *POT1* PV underwent a surveillance program according to the current recommendations [1]. The individual III.9 (Fig. 1) was diagnosed with low grade glioma at age 50, after a brain MRI performed as part of the surveillance program. No other tumours were detected in other individuals. However, itis important to note that most of them are young and they are under this surveillance program for one to four years only.

This study has some limitations that are important to mention, the small number of individuals tested in families B and C, the fact that the test is not carried out consecutively on all family members, but only on the ones with indication and willing to do it. Also, cancer history of relatives, mainly regarding the first families’ generations, was based on family-reporting information, which may contribute to less accurate data.

Despite these, the wide pattern of cancers observed in the three families suggests a larger POT1-TPD spectrum than the one previously described [1]. The presence of a *POT1* PV, its penetrance, genetic modifiers and other external factors are possible causes for the different phenotypes observed. We estimate that POT1-TPD is probably underdiagnosed with the current recommendation criteria prompting to search for germline *POT1* PV.

Regarding the age to start testing patients and relatives, our findings are in accordance with the current recommendations, since all individuals in these families developed cancer in their adulthood [1].

According to our findings, we consider that it would be important to discuss the inclusion of all types of sarcomas in the screening criteria for *POT1* mutations. Also, the observation of early prostate cancer in these families may be a point to discuss with patients, namely the role of prostate cancer screening, on an individual basis and taking into account patient family history. Current recommendations already recommend a whole-body MRI for screening of cardiac angiosarcoma, which is the ideal screening exam for other types of sarcoma too [1]. The main recommendations suggest a surveillance protocol similar to Li–Fraumeni-like patients, with whole-body MRI and in POT1 carriers not fulfilling these criteria, MRI should be considered depending on personal and family history of non-cutaneous, non-brain malignancies [1, 20]. This is an important topic, since it is estimated that POT1 PV carriers have 6 times more risk of developing a sarcoma than POT1 wild type individuals [13]. A comprehensive physical exam and a complete blood count are already proposed to screen for LLC and will be useful in screening other haematological malignancies as well. We also hypothesise the clinical utility of earlier start of prostate cancer screening with annual PSA dosage from age 40-45 years, or 10 years before the youngest case diagnosed in the family, on an individual basis, as discussed before, since it is a non-invasive and non-expensive test. Another point to consider in surveillance programs would be behavioural measures such us smoking cessation, avoidance of smoke and occupational carcinogens, eating a healthy diet and exercising regularly.

Despite the limitations of this study, we conclude that it suggests that *POT1* germline PV are associated with a broader spectrum of hereditary cancer than the previously described POT1-TPD. Since some of the reported tumours are also common in the general population, a clear definition of POT1 tumour spectrum is hard to make. Nonetheless, our observations align with recent studies also reporting sarcoma (and cardiac angiosarcoma), CRC and PTC associated to *POT1* PV [11, 13, 14, 16–19]. Moreover, the cases of leukaemia described in these *POT1* carrier families are in accordance with more recent data about the link between *POT1* PV and the higher risk of B/T-cell lymphoproliferative and myeloproliferative diseases [6]. These findings are important to better understand the implications of *POT1* PV, its prevalence and penetrance. Furthermore, findings of a broader spectrum of diseases related to POT1-TPD should be considered for future guidelines, not only about the testing criteria for *POT1* PV, but also to adapt the surveillance program to these other malignancies within the POT1-TPD (mainly sarcomas). Further studies on *POT1* PV, their penetrance and associated types of cancers are needed, especially prospective trials with large cohorts of patients, in order to improve the knowledge of cancer genetic mechanisms, *POT1* cancer spectrum and also the genetic counselling for these patients and their families.

## Data Availability

*POT1* variants mentioned in this article (p. Arg117Cys, p. Ile78Thr and p. Arg273Gln) were submitted to ClinVar (Variants ID: SCV004805901, SCV004806485, SCV004805248, respectively).
